# Molecular Characterization, Virulence Profiling, and Antimicrobial Susceptibility of *Listeria monocytogenes* Isolated from Smoked Fish in Poland: A Preliminary Study

**DOI:** 10.3390/foods15081406

**Published:** 2026-04-17

**Authors:** Zuzanna J. Strzałkowska, Ewa D. Domańska, Karolina Wódz, Magdalena Kizerwetter-Świda, Dorota Chrobak-Chmiel, Tomasz Nowak, Piotr Kwieciński, Elżbieta Rosiak, Kamil Stańczak, Joanna Pławińska-Czarnak

**Affiliations:** 1 Department of Preclinical Sciences, Institute of Veterinary Medicine, Warsaw University of Life Sciences-SGGW, Ciszewskiego Str. 8, 02-786 Warsaw, Poland; zuzanna_strzalkowska@sggw.edu.pl (Z.J.S.); domanskaewa717@gmail.com (E.D.D.); magdalena_kizerwetter_swida@sggw.edu.pl (M.K.-Ś.); dorota_chrobak-chmiel@sggw.edu.pl (D.C.-C.);; 2 Laboratory of Molecular Biology, Vet-Lab Brudzew, Turkowska 58c, 62-720 Brudzew, Poland; karolina.wodz@labbrudzew.pl (K.W.); tomasz@labbrudzew.pl (T.N.); piotr.kwiecinski@mail.com (P.K.); 3 Department of Food Gastronomy and Food Hygiene, Institute of Human Nutrition Sciences, Warsaw University of Life Sciences-SGGW, Nowoursynowska Str. 166, 02-787 Warsaw, Poland; elzbieta_rosiak@sggw.edu.pl

**Keywords:** *Listeria monocytogenes*, LIPI-1, PFGE, antimicrobial susceptibility, fish

## Abstract

*Listeria monocytogenes* remains a major foodborne pathogen associated with ready-to-eat (RTE) products, including smoked fish. This study investigated the occurrence, molecular characteristics, virulence gene profiles, and antimicrobial susceptibility of *L. monocytogenes* isolated from retail smoked fish in Poland. A total of 46 samples (cold- and hot-smoked products) collected from 15 producers and five retail chains were analyzed using ISO 11290-1:2017 for qualitative detection and ISO 11290-2:2017 for enumeration. *Listeria* spp. were detected in 5/46 samples (10.9%), including 4 isolates confirmed as *L. monocytogenes* (8.7%). All positive samples originated from cold-smoked salmon, with a prevalence of 4/13 (30.8%) in this product category. The quantitative analysis indicated that contamination levels in all positive samples were below 100 CFU/g. Molecular serogrouping and multiplex PCR demonstrated the presence of key virulence-associated genes, including *hlyA*, *prfA*, *plcB*, and *actA*, consistent with potentially pathogenic profiles. Pulsed-field gel electrophoresis (PFGE) revealed clustering of isolates, indicating genetic relatedness among strains obtained from different retail sources. Antimicrobial susceptibility testing using the MICRONAUT system showed that all *L. monocytogenes* isolates were susceptible to first-line therapeutic agents, including ampicillin and penicillin, according to EUCAST/CLSI criteria. Although contamination levels were low and isolates remained susceptible to clinically relevant antimicrobials, the detection of virulence-associated strains in RTE smoked fish highlights the need for continuous monitoring and strict hygienic control in the production and retail chain. These findings contribute to regional surveillance data on *L. monocytogenes* in smoked fish products in Poland.

## 1. Introduction

*Listeria monocytogenes* remains one of the most severe foodborne pathogens, consistently associated with the highest hospitalization and case-fatality rates among zoonoses in Europe [1,2]. While in 2022, 2.770 confirmed human cases were reported in the European Union, the latest surveillance data from 2024 to 2025 indicate a persistent challenge, with incidence rates in Poland remaining significant [1,2]. The disease primarily affects high-risk groups, including pregnant women, newborns, the elderly, and immunocompromised individuals, in whom infection may result in life-threatening conditions such as septicemia, meningitis, or spontaneous abortion [2,3].

From a public health and regulatory perspective, 1 July 2026 marks an important date for the food industry, as Commission Regulation (EU) 2024/2895 will apply from that date, introducing updated microbiological criteria for *L. monocytogenes* in ready-to-eat (RTE) foods. This new legislative framework significantly tightens the safety criteria for *L. monocytogenes* in ready-to-eat (RTE) foods [4]. While historical limits allowed up to 100 CFU/g during shelf-life, the current mandate enforces an “absence in 25 g” criterion throughout the entire shelf-life for products where growth is possible, unless the food business operator can provide robust scientific evidence that the 100 CFU/g limit will not be exceeded [5,6]. This shift is a direct response to the rising number of outbreaks, where fish and fishery products have been repeatedly implicated [2,7].

Globally, fish constitute an essential component of human diets and represent one of the most important sources of high-quality animal protein and micronutrients supporting sustainable food systems. Between 2020 and 2022, global apparent fish consumption averaged 20.4 kg per capita, and is projected to reach 21.2 kg by 2034, with aquaculture expected to account for nearly 60% of total fish supply [8]. In Europe, per capita fish consumption remains relatively stable at around 23.5 kg, with salmon and cod being the dominant species [8,9]. In Poland, per capita consumption is lower (14.3 kg), yet the country remains the largest European processor and exporter of smoked fish products, mainly based on imported Atlantic salmon—accounting for about 70% of total EU production [10]. This emphasizes the importance of monitoring *L. monocytogenes* in smoked fish as a critical step in ensuring food safety and consumer protection.

**Figure 1:**
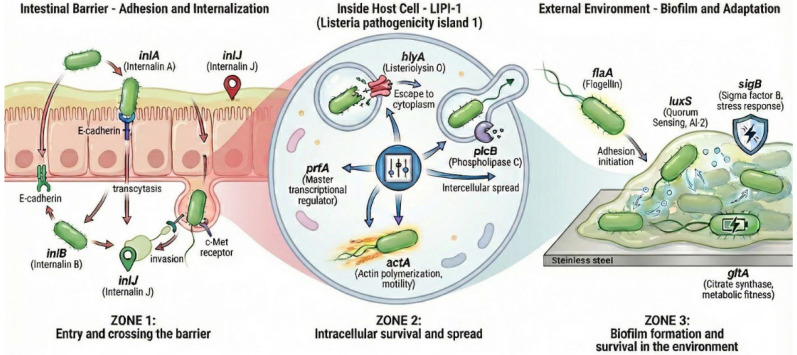
Schematic representation of the *L. monocytogenes* life cycle, highlighting key molecular determinants of host virulence and environmental adaptation across sequential stages.

Although *L. monocytogenes* is primarily monitored in foods by culture-based detection and enumeration, linking phenotypic findings to pathogen biology benefits from a structured view of the infection cycle and persistence traits. For molecular target selection, the infection biology of *L. monocytogenes* may be conceptualized as a sequence of functionally distinct stages: (i) adhesion to and invasion of the intestinal barrier, (ii) intracellular survival with subsequent cell-to-cell dissemination, and (iii) persistence under food-processing conditions through stress tolerance and biofilm-associated traits. These stages are underpinned by well-characterized genetic determinants, including internalins (*inlA*, *inlB*, *inlJ*), the LIPI-1 virulence regulon (*prfA*, *hlyA*, *plcB*, *actA*), and factors linked to motility, quorum sensing, stress response, and metabolic fitness (*flaA*, *luxS*, *sigB*, *gltA*) (Figure 1).

The scheme illustrates three functional stages: (Zone 1) intestinal barrier adhesion and invasion mediated by internalins (*inlA*, *inlB*, *inlJ*); (Zone 2) intracellular survival and cell-to-cell spread regulated by the LIPI-1 cluster (*prfA*, *hlyA*, *plcB*, *actA*); and (Zone 3) environmental persistence and biofilm formation involving motility, quorum sensing, stress adaptation, and metabolic fitness determinants (*flaA*, *luxS*, *sigB*, *gltA*). Created by the authors.

This conceptual structure facilitates an integrated interpretation of isolate virulence potential alongside attributes relevant to survival and dissemination within RTE food chains.

The growing concern regarding antimicrobial resistance (AMR) in *L. monocytogenes* from both food and clinical sources further underscores the importance of continuous surveillance [11,12,13]. Although most isolates remain susceptible to first-line therapeutics, emerging resistance patterns reported in Europe, including Poland, highlight the need to monitor susceptibility profiles in parallel with molecular characterization [12,14]. Despite ongoing surveillance programs, data regarding the prevalence, molecular diversity, and pathogenic potential of *L. monocytogenes in* retail smoked fish in Poland remain limited [2,11,12].

Therefore, this preliminary study aimed to assess the occurrence, molecular serogroups, genetic relatedness using pulsed-field gel electrophoresis (PFGE), virulence-associated gene profiles (including LIPI-1 and internalins), and antimicrobial susceptibility of *L. monocytogenes* isolates recovered from retail smoked fish in Poland. From a food safety perspective, the work was designed as a surveillance-oriented assessment of ready-to-eat (RTE) smoked fish, with particular emphasis on combining quantitative microbiological findings with virulence-associated characteristics and phenotypic antimicrobial susceptibility of the recovered isolates. This integrated characterization provides relevant information for assessing the risk posed by *L. monocytogenes* in smoked fish, supports producers in meeting current EU microbiological criteria, and may help identify potential routes of persistent contamination in the smoked fish supply chain.

## 2. Materials and Methods

### 2.1. Sample Collection

A total of 46 retail smoked fish products were collected from fifteen fish producers and five major retail chains located in the Mazowieckie Voivodeship (central Poland) as part of a cross-sectional retail survey. All products were purchased at the retail level as ready-to-eat (RTE) items intended for direct consumption without further heat treatment.

The analyzed products represented different fish species and smoking types, including both cold-smoked and hot-smoked products. Samples were numbered consecutively in the order of analysis (Fish1–Fish46) and, for consistency throughout this manuscript, are reported using the corresponding codes R1–R46. All samples were within their declared shelf life at the time of analysis, transported to the laboratory under refrigerated conditions (4 ± 1 °C), and analyzed within 6 h of purchase. A summary of the analyzed samples and their characteristics is presented in Table 1.

### 2.2. Listeria spp. Isolation and Identification

#### 2.2.1. Microbiological Detection of *L. monocytogenes*

Qualitative detection of *L. monocytogenes* was performed in accordance with ISO 11290-1:2017 [15]. Briefly, 25 g of each sample was subjected to primary enrichment in Half-Fraser broth and secondary enrichment in Fraser broth under (BioMaxima, Lublin, Poland) standard incubation conditions. Enrichment cultures were streaked onto ALOA (BioMaxima, Lublin, Poland) and Oxford agar (Oxoid, Basingstoke, UK) for selective isolation.

Presumptive *Listeria* colonies were purified and subjected to microscopic examination, hemolysis assessment, and biochemical identification using the Listeria 18R system (Liofilchem, Roseto degli Abruzzi, Italy) and the VITEK^®^ 2 COMPACT system (bioMérieux, Craponne, France). Confirmed isolates were stored in BHI broth supplemented with 20% glycerol at −20 °C until further molecular analysis.

#### 2.2.2. Enumeration of *L. monocytogenes*

Quantitative determination of *L. monocytogenes* was performed in accordance with ISO 11290-2:2017 [16]. Briefly, 25 g of each sample was homogenized with 225 mL of Buffered Peptone Water (BPW) (BioMaxima, Lublin, Poland) in sterile stomacher bags (Chemilab, Tarnobrzeg, Poland) for 2 min.

Serial tenfold dilutions were prepared in sterile diluent. For enumeration, 1 mL of the appropriate dilution was surface-plated onto Agar Listeria according to Ottaviani and Agosti (ALOA) plates (90 mm diameter; BioMaxima, Lublin, Poland) in duplicate for each dilution level. Plates were incubated at 37 ± 1 °C for 24 ± 2 h and extended to 48 h if necessary.

Colonies showing typical *Listeria* morphology were counted, and up to five representative colonies per sample were purified and confirmed as described in Section 2.2.1. Results were expressed as colony-forming units per gram (CFU/g) in accordance with ISO 7218:2024 [17]. The limit of quantification (LoQ) of the method was 10 CFU/g.

#### 2.2.3. Biochemical Identification of *L. monocytogenes*

Biochemical identification of presumptive *L. monocytogenes* isolates was performed using the Listeria 18R identification system (Liofilchem, Roseto degli Abruzzi, Italy) and the VITEK^®^ 2 COMPACT automated system (bioMérieux, Craponne, France), according to the manufacturers’ instructions.

The identification procedure included evaluation of carbohydrate fermentation profiles (rhamnose, xylose, and mannitol), hemolytic activity on blood agar, catalase production, and assessment of bacterial motility at 25 ± 1 °C. Identification results obtained using the Listeria 18R system were verified with the VITEK^®^ 2 COMPACT system to ensure consistency.

Each analytical batch included appropriate control strains. *L. monocytogenes* ATCC 19111 was used as the positive control, and *L. innocua* ATCC 33090 served as the negative control.

#### 2.2.4. DNA Extraction and Quality Assessment

Genomic DNA was extracted from pure bacterial cultures using the BacBreaker reagent in combination with the MagnifiQ™ 1 Genomic DNA Instant Kit (A&A Biotechnology, Gdańsk, Poland), according to the manufacturer’s instructions. DNA purification was carried out using the Auto-Pure Mini automated system (A&A Biotechnology, Gdańsk, Poland).

DNA concentration and purity were assessed spectrophotometrically using a NanoDrop spectrophotometer (Thermo Fisher Scientific, Waltham, MA, USA) by measuring absorbance at 260/280 nm and 260/230 nm. Samples with an A260/A280 ratio of 1.8–2.0 and an A260/A230 ratio above 1.8 were considered suitable for downstream molecular analyses.

When necessary, DNA samples were diluted with nuclease-free water to obtain a working concentration of 50–100 ng/µL. For PCR amplification, 1–2 µL of template DNA was used in a final reaction volume of 25 µL. Extracted DNA was stored at −20 °C until further analysis.

#### 2.2.5. Molecular Identification and Serotyping of *L. monocytogenes*

Definitive species-level identification of the presumptive *L. monocytogenes* isolates was performed via a specific PCR assay targeting the *lmo2234* gene (420 bp), a highly conserved marker unique to this species [18]. Following species confirmation, molecular serogrouping was conducted using a multiplex polymerase chain reaction (PCR) assay described by Doumith et al. (2004) [19,20], with minor modifications. This multiplex method allows for the differentiation of the four major molecular serogroups: IIa (1/2a, 3a), IIb (1/2b, 3b, 7), IIc (1/2c, 3c), and IVb (4b, 4d, 4e), based on the simultaneous detection of the *lmo0737*, *lmo1118*, *orf2110*, *orf2819*, and the genus-specific *prs* genes.

For both the singleplex (*lmo2234*) and multiplex serogrouping assays, each PCR (25 µL) contained 12.5 µL of 2× DreamTaq Green PCR Master Mix (Thermo Fisher Scientific, Waltham, MA, USA), specific primers at a final concentration of 0.2 µM each, 2 µL of template DNA (50–100 ng/µL), and nuclease-free water to reach the final volume. Detailed primer sequences, target genes, and expected amplicon sizes are provided in Supplementary Table S1.

#### 2.2.6. Detection of Virulence and Biofilm-Associated Genes

To assess the virulence potential and environmental persistence of the analyzed *L. monocytogenes* isolates, a panel of genes associated with host cell invasion (*inlA*, *inlB*, *inlJ*), intracellular survival and spread (*hlyA*, *prfA*, *plcB*, *actA*), stress adaptation (*sigB*), quorum sensing (*luxS*), motility (*flaA*), and metabolic fitness (*gltA*) was selected. The biological roles of the investigated determinants are illustrated schematically in Figure 1.

PCR amplifications were performed using DreamTaq Green PCR Master Mix (Thermo Fisher Scientific, Waltham, MA, USA) in a final reaction volume of 25 µL. Primers were used at a final concentration of 0.2 µM each per reaction. For multiplex PCR assays, 2 µL of template DNA was added per reaction, whereas 1 µL was used for singleplex PCR assays. Genomic DNA was standardized to 50–100 ng/µL prior to amplification.

Negative controls (no-template controls, NTCs) were included in each PCR run by replacing template DNA with nuclease-free water. Previously described reference strains were included as positive controls for the respective target genes.

Amplification was performed using a ProFlex PCR System thermal cycler (Applied Biosystems, Thermo Fisher Scientific, Waltham, MA, USA). Thermal cycling conditions were optimized according to primer melting temperatures and expected amplicon sizes. Detailed cycling parameters for each assay are provided in Table 2. Primer sequences were adopted from previously published assays [21,22,23,24,25,26,27,28,29,30,31,32,33,34,35,36,37,38,39].

Detailed thermal cycling programs for the individual PCR assays are summarized in Table 2 below. To ensure assay validity, the following reference strains and well-characterized laboratory isolates were used as positive controls for the respective target genes: *L. monocytogenes* ATCC 15313, ATCC 7644, and ATCC 19111.

PCR products were separated on 2% agarose gels, stained with SimplySafe™ DNA Stain, visualized under UV illumination, and documented using a ChemiDoc MP Imaging System (Bio-Rad Laboratories, Hercules, CA, USA).

#### 2.2.7. Pulsed-Field Gel Electrophoresis (PFGE)

PFGE analysis was performed according to the standardized PulseNet protocol for *L. monocytogenes* subtyping, with minor modifications [3].

Briefly, *L. monocytogenes* isolates were cultured on Columbia blood agar (GRASO Biotech, Starogard Gdański, Poland) at 37 °C for 17 ± 1 h. Bacterial cells were resuspended in PBS buffer and the cell density was adjusted to 3.5 in McFarland standard using a densitometer (bioMérieux, Craponne, France). Then 150 μL of bacterial suspension was mixed in a ratio 1:1 with molten 2% agarose (BioRad, Warsaw, Poland) and agarose discs were formed. After solidification, the agarose discs were incubated with a lysozyme (100 mg/mL) and RNase (10 mg/mL) at 37 °C overnight. The next overnight incubation was carried out with proteinase K (20 mg/mL) at 50 °C. Finally, DNA was digested with ApaI (EURx, Gdańsk, Poland) and the DNA fragments were separated in 1% agarose (BioRad, Warsaw, Poland) in CHEF DRII system (BioRad, Warsaw, Poland) according to standard protocol. After staining with ethidium bromide (0.5 μg/mL) gel images were documented and analyzed by BioNumerics v. 7.0 (Applied Maths).

The ApaI-PFGE profiles were analyzed by the unweighted pair group method (UPGMA) and Dice similarity coefficient with optimization set at 0.5% and position tolerance 1.5%. Isolates were clustered together when restriction profiles shared at least 80% similarity. The reference *L. monocytogenes* ATCC 13932 strain was used as a control.

#### 2.2.8. Antimicrobial Resistance Testing

Phenotypic antimicrobial susceptibility of *L. monocytogenes* isolates was determined by minimum inhibitory concentration (MIC) testing using 96-well MICRONAUT plates (MERLIN Diagnostika GmbH, Bremen, Germany), according to the manufacturer’s instructions. The antimicrobial panel included representatives of β-lactams, aminoglycosides, macrolides, tetracyclines, fluoroquinolones, lincosamides, polymyxins, phenicols, and trimethoprim-sulfamethoxazole. A detailed list of antimicrobial agents and concentration ranges is provided in Supplementary Table S5.

MIC values were interpreted in accordance with the European Committee on Antimicrobial Susceptibility Testing (EUCAST, version 14.0) and the Clinical and Laboratory Standards Institute (CLSI M45) guidelines, where applicable; the corresponding breakpoints/interpretive criteria used in this study are provided in Supplementary Table S5. Antimicrobial agents lacking established clinical breakpoints for *L. monocytogenes* were included for epidemiological purposes only and were not used for clinical resistance categorization [46,47].

## 3. Results

### 3.1. Prevalence of L. monocytogenes

Among the 46 analyzed smoked fish samples, presumptive *Listeria* spp. were detected in 5 samples (10.9%) based on characteristic growth on selective media. Subsequent biochemical identification and PCR confirmation revealed that 4 isolates were *L. monocytogenes*, while one isolate was identified as *L. innocua*. This corresponds to an overall prevalence of *L. monocytogenes* of 8.7% (4/46).

All confirmed *L. monocytogenes* isolates originated from cold-smoked, sliced Atlantic salmon. The prevalence among salmon products was 16.7% (4/24), and specifically 30.8% (4/13) among cold-smoked salmon samples. The positive samples represented different production batches and originated from two of the fifteen producers included in the study.

No *L. monocytogenes* was detected in hot-smoked fish products or in smoked products derived from other fish species.

### 3.2. Enumeration of L. monocytogenes

According to Regulation (EC) No 2073/2005 numbers of *L. monocytogenes* in none of the samples exceeded the limit of 100 CFU/g required for RTE food products in the presence of shelf-life [7]. In 42 cases (93.5%) no presumptive *Listeria* bacterial growth was obtained. Furthermore, only one of the four positive cases (from cold-smoked salmon) showed *L. monocytogenes* characteristic colonies (2.2%); the colony count was <100 CFU/g. The level of contamination was 1.5 × 10 CFU/g.

### 3.3. Biochemical Identification of L. monocytogenes

Biochemical identification using the Listeria System 18R (Liofilchem) confirmed isolates R10, R18, R31, and R46 as *L. monocytogenes*, whereas isolate R14 was identified as *L. innocua*. Subsequently, biochemical profiling performed with the VITEK^®^ 2 COMPACT system classified the same isolates (R10, R18, R31, and R46) as *L. monocytogenes* and confirmed R14 as *L. innocua*, demonstrating complete concordance between both identification approaches. The prevalence of detected *Listeria* spp. is presented in Table 3. Both systems showed identical key biochemical characteristics typical of *L. monocytogenes*, including positive reactions for catalase (CAT), methyl red (MR), esculin hydrolysis (ESC), glucose fermentation (GLU), and Voges-Proskauer (VP). In contrast, negative reactions were observed for urease (URE), hydrogen sulfide production (H_2_S), indole production (IND), and nitrate reduction (NIT). Additionally, isolate R14 displayed a biochemical profile characteristic of *L. innocua*, with positive reactions for xylose fermentation (XYL), saccharose utilization (SAC), and lactose fermentation (LAC) in the VITEK^®^ 2 COMPACT system, which are not typical for *L. monocytogenes*. These features were consistent with the identification from the Listeria System 18R, further confirming the species assignment. Detailed biochemical reaction profiles and species identification results obtained using the VITEK^®^ 2 COMPACT system and Listeria System 18R are presented in the Supplementary Materials Tables S2 and S3.

### 3.4. Molecular Species Confirmation and Serogrouping

The biochemical identification of the isolates was definitively confirmed using species-specific PCR. Amplification of the *lmo2234* marker yielded the expected specific amplicon in all four isolates previously identified as *L. monocytogenes* (R10, R18, R31, and R46), whereas the *L. innocua* isolate R14 and the no-template control remained negative (Figure S1).

Following species confirmation, molecular serogrouping using multiplex PCR targeting lineage-specific markers [20] revealed the presence of two distinct molecular serogroups among the *L. monocytogenes* isolates. Isolates R10 and R31 were classified as serogroup IIa, while isolates R18 and R46 were assigned to serogroup IIc. The isolate R14 exhibited only the genus-specific prs marker, consistent with its identification as *L. innocua* (Figure S2).

A summary of biochemical identification, molecular confirmation, and serogrouping results is presented in Table 4.

Representative gel images are provided in the Supplementary Materials (Figures S1 and S2).

### 3.5. Virulence and Biofilm-Associated Gene Profiling

PCR-based screening was performed to determine the presence of genes associated with the *Listeria* Pathogenicity Island 1 (LIPI-1), the internalin family, stress response, quorum sensing, motility, and metabolic fitness.

All four *L. monocytogenes* isolates (R10, R18, R31, and R46) carried the investigated LIPI-1 genes (*prfA*, *hlyA*, *plcB*, and *actA*) as well as the internalin genes (*inlA*, *inlB*, and *inlJ*), indicating a conserved virulence-associated profile. In contrast, the *L. innocua* isolate R14 lacked the complete virulence profile and showed only a faint non-specific band at the *hlyA* locus.

In addition, genes associated with environmental adaptation and persistence (*sigB*, *luxS*, *flaA*, and *gltA*) were detected in all *L. monocytogenes* isolates. The same set of environmental fitness-related markers was also detected in the *L. innocua* isolate R14, consistent with their broader role in stress tolerance, quorum sensing, motility, and metabolic adaptation rather than species-specific virulence.

A complete overview of gene distribution is presented in Table 5.

Representative PCR results are provided in the Supplementary Materials (Figures S3–S6).

### 3.6. Pulsed-Field Gel Electrophoresis (PFGE) Results of L. monocytogenes Strains

Pulsed-field gel electrophoresis (PFGE) analysis using the ApaI restriction enzyme was performed for all four *L. monocytogenes* isolates. The obtained macrorestriction profiles were analyzed using the Dice similarity coefficient and the unweighted pair group method with arithmetic mean (UPGMA).

At an 80% similarity threshold, two distinct clusters were identified. Isolates R18 and R46 demonstrated 92.9% similarity and formed one cluster, whereas isolates R10 and R31 showed 88.9% similarity and formed a second cluster.

The isolates included in each cluster originated from different production batches. No identical PFGE profiles (100% similarity) were observed among the analyzed strains.

Due to the limited number of isolates included in this study, the PFGE results describe genetic relatedness among the strains but do not allow conclusions regarding contamination routes, persistence within processing environments, or epidemiological linkage.

**Figure 2:**
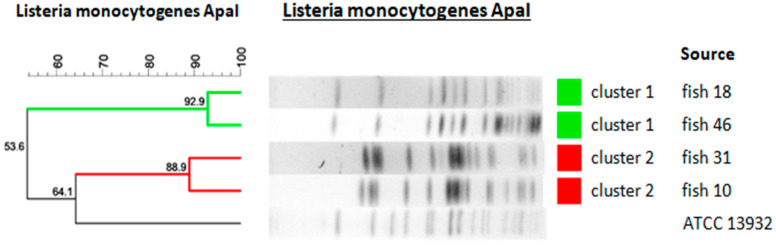
Dendrogram of PFGE profiles from examined smoked fish isolates.

Figure 2 shows a dendrogram illustrating the genetic relatedness of *L. monocytogenes* isolates recovered from retail smoked fish, based on ApaI-PFGE restriction profiles. The analysis was performed using the unweighted pair group method with arithmetic mean (UPGMA) and the Dice similarity coefficient, with optimization set at 0.5% and position tolerance at 1.5%. The vertical line indicates the 80% similarity threshold used to define the two main clusters (Cluster 1 and Cluster 2). The reference strain *L. monocytogenes* ATCC 13932 was included as an outgroup control.

### 3.7. Antibiotic Resistance in Isolated L. monocytogenes Strains

The phenotypic antimicrobial susceptibility of the recovered *Listeria* isolates was determined by evaluating the Minimum Inhibitory Concentrations (MICs) using the broth microdilution method. Based on the established clinical breakpoints, the four *L. monocytogenes* strains (R10, R18, R31, and R46) and the atypical *L. innocua* strain (R14) exhibited a pan-susceptible profile to all evaluated antimicrobial agents.

Specifically, isolates R10, R18, R31, R46, and R14 demonstrated uniform susceptibility to standard first-line therapeutics used for listeriosis treatment, including benzylpenicillin (MIC ≤ 1 mg/L) and amoxicillin (susceptibility inferred from benzylpenicillin). Furthermore, all five individual strains were susceptible to erythromycin (MIC ≤ 1 mg/L) and trimethoprim-sulfamethoxazole (MIC ≤ 0.06 mg/L, expressed as the trimethoprim concentration). No acquired resistance phenotypes or multi-drug resistance (MDR) profiles were observed among any of the isolates recovered from the smoked fish samples.

## 4. Discussion

According to EFSA data for 2022, 7.1% of ready-to-eat (RTE) fish and fishery products in the European Union tested positive for *L. monocytogenes*. In Poland, *L. monocytogenes* was reported in 10% of the examined samples [2]. In the present study, the overall prevalence of *L. monocytogenes* was lower than that reported in several European surveys; however, the proportion of positive cold-smoked salmon samples was comparatively higher. A recent systematic review and meta-analysis of fish, fish products, and fish-processing environments further highlights the relevance of this product category as a vehicle for *Listeria* spp. in the food chain [48].

Domínguez et al. [49]. detected *Listeria* spp. in 47.6% of smoked fish samples and *L. monocytogenes* in 22.3% of smoked fish products in Spain. Specifically, cold-smoked vacuum-packed and unpacked salmon samples were contaminated in 17.7% and 28.5%, respectively (overall 21.2% of cold-smoked salmon products). In Italy, *L. monocytogenes* was detected in 18.5% of smoked fish samples tested immediately upon receipt at the laboratory and in 20.2% of all samples when testing was repeated at the end of shelf life [50]. In Lithuania, culture-based screening indicated the presence of *Listeria* spp. in 32.5% of cold-smoked salmon samples; multiplex PCR confirmation was obtained for 31.3% of cases. *L. monocytogenes* was detected in 23.1%, *L. innocua* in 1.9%, and other species (*L. ivanovii*, *L. seeligeri*, and *L. welshimeri*) in 6.1% of products; salmon belly flaps were the most frequently contaminated commodity [51]. In Poland, the proportion of positive cold-smoked salmon samples reported in studies conducted in 2017 and 2020 was 18.4% and 15.7%, respectively, which is lower than observed in the present study [11,52]. Notably, cold-smoked rainbow trout examined in 2017 showed no evidence of contamination [52]. Moreover, Szymczak et al. [53], assessing RTE foods in Poland, detected *Listeria* spp. in two of five smoked fish samples. Hot smoking in commercial food production reduces microbial contamination, similarly to pasteurization. However, post-process recontamination may occur during handling, slicing, and packaging, for example due to inadequate hygienic practices (e.g., insufficient protective clothing), unsanitary environmental conditions (including insects and dust), and suboptimal processing procedures [54]. Domínguez et al. [49] reported *L. monocytogenes* in both vacuum-packed and unpacked products, suggesting that contamination can occur before packaging and/or during post-smoking processing steps. In addition, raw fish can serve as an upstream source of *Listeria* spp., as demonstrated in recent Polish data on isolates from raw fish [55].

None of the samples analyzed in the present study exceeded the acceptable limit of 100 CFU/g for *L. monocytogenes*. In Poland, the proportion of smoked fish samples exceeding 100 CFU/g in 2017 was also low (approximately 2%), although the highest reported contamination level reached 7.6 × 10^3^ CFU/g [52]. Similarly, in the study by Szymczak et al. [53], one smoked fish sample containing *L. monocytogenes* exceeded 100 CFU/g.

In Spain, *L. monocytogenes* was detected at levels <100 CFU/g in 10.6% of smoked fish samples and at 100–1000 CFU/g in another 10.6%; only 2 products (1.2%) contained >1000 CFU/g [49]. In Italy, 18.5% of smoked fish products tested positive upon arrival at the laboratory, yet only 2.3% exceeded 100 CFU/g, and almost all positive samples were smoked salmon; the maximum reported level was 1 × 10^6^ CFU/g [50]. The authors also suggested that contamination levels may vary between manufacturing plants and reported an association between PFGE pulsotypes and product manufacturer, indicating that persistence in processing environments and contamination of final products may be linked to plant-specific hygiene practices [50]. Consistent with this, targeted control strategies in slicing/packaging areas and enhanced environmental monitoring are considered critical to reduce *L. monocytogenes* contamination in RTE smoked fish. Pulsed-field gel electrophoresis (PFGE) remains a useful method for assessing relatedness among isolates, including during outbreak investigations, although higher-resolution approaches, particularly whole genome sequencing (WGS), are currently considered the standard for high-resolution typing of *L. monocytogenes* [11,56,57]. In the present study, PFGE was applied as a complementary rather than a stand-alone approach, alongside serogrouping, virulence-associated gene profiling, and MIC-based antimicrobial susceptibility testing. From the perspective of RTE food safety, this combined approach is relevant because it links contamination data with pathogenic potential and the observed antimicrobial susceptibility phenotype. Moreover, growth potential under different refrigerated storage conditions has been demonstrated for RTE fish products, emphasizing that low initial contamination does not preclude higher levels at the end of shelf life [58,59].

By contrast, *L. monocytogenes* was not detected in smoked or smoked-dried fish samples examined in Benin [54], highlighting that prevalence can vary substantially across regions and production systems.

Molecular profiling indicated that isolate R14 belonged to a non-*monocytogenes Listeria* species. This interpretation is supported by amplification of prs, a genus-level marker for *Listeria* [20], together with the absence of the *L. monocytogenes*-specific target *lmo2234* and the internalin genes (*inlA*, *inlB*, *inlJ*) typically associated with pathogenic *L. monocytogenes* lineages and host cell invasion [60]. In addition, R14 was negative for the serogrouping markers (*lmo0737*, *lmo1118*, *ORF2819*, *ORF2110*) used in the Doumith multiplex PCR scheme [20]. Notably, LIPI-1 multiplex PCR produced a weak band only at the *hlyA* locus, whereas *prfA*, *plcB*, and *actA* were not detected. Such faint amplification in non-*monocytogenes* isolates is most plausibly attributable to non-specific primer binding or cross-reactivity with homologous hemolysin-related sequences described in other *Listeria* spp. [61,62]. Because a complete LIPI-1 profile was not observed, the weak *hlyA* signal should not be interpreted as evidence of a functional virulence island.

Finally, amplification of *sigB* and *luxS* indicates the presence of conserved stress-response and quorum-sensing/biofilm-associated determinants, supporting the potential of non-pathogenic *Listeria* spp. to persist in food-processing environments alongside *L. monocytogenes*. Recent studies focusing on isolates from fish and fish-industry environments provide further evidence that virulence- and persistence-associated traits frequently co-occur in food-chain strains [63]. The predominance of serogroups IIa and IIc observed in this study is consistent with previous reports indicating their association with food processing environments and their ability to persist in production facilities [63,64,65]. These serogroups are frequently isolated from RTE foods and are considered well-adapted to food-related niches, which may facilitate long-term contamination of processing environments and subsequent contamination of final products.

According to Di Ciccio et al. [64], contamination of cold-smoked salmon is more likely to occur during processing than to originate from raw fish. In the present study, PFGE revealed high similarity (>80%) between isolates originating from different production facilities, while isolates obtained from the same source were occasionally less related (below the applied similarity cut-off). This pattern is compatible with the circulation of closely related strains across the supply chain and/or the presence of more than one *L. monocytogenes* strain within a single production setting.

The coexistence of multiple strains may increase the likelihood of consumer exposure to genetically distinct *Listeria* populations and may complicate outbreak investigations [56]. Importantly, the recovery of *L. monocytogenes* from production-associated products can indicate shortcomings in hygiene and sanitation; however, the persistence of *Listeria* spp. is also supported by their ability to form biofilms and tolerate environmental stressors, which makes eradication challenging. Therefore, intensified cleaning, equipment decontamination, and environmental monitoring-particularly at slicing and packaging steps-remain critical control measures [65,66]. This interpretation is consistent with reports from fish-processing plants describing persistent versus sporadic *L. monocytogenes* populations and the challenges of environmental control in salmon-associated production settings [67,68,69].

*Listeria monocytogenes* infections are typically treated with β-lactams (e.g., ampicillin or penicillin), often combined with an aminoglycoside (e.g., gentamicin), whereas trimethoprim–sulfamethoxazole is commonly considered an alternative option. Although *L. monocytogenes* is intrinsically non-susceptible to cephalosporins, acquired resistance to clinically relevant agents has been increasingly reported in isolates from food, the environment, and human cases, underscoring the importance of ongoing surveillance across geographic regions [12,13,14,52,70,71]. Comparable assessments of virulence and antimicrobial susceptibility in RTE foods and salmon-based products have been reported in other European and international settings [72,73].

In the present study, all *L. monocytogenes* isolates displayed a homogeneous, fully susceptible phenotype in the tested panel. This aspect is particularly important because genomic data may indicate resistance potential, whereas MIC testing reflects the directly observed phenotypic expression of susceptibility. From a food-safety perspective, this finding suggests a low likelihood that the investigated products currently serve as a reservoir for acquired antimicrobial resistance in *L. monocytogenes*. Nevertheless, continued monitoring remains warranted, particularly in RTE foods with long refrigerated shelf life.

While serogrouping and PFGE provided useful preliminary insights into the genetic relatedness and potential epidemiological significance of the isolates, these approaches do not allow precise clonal characterization. In particular, the lack of multilocus sequence typing (MLST) or whole-genome sequencing (WGS) data precludes assignment of isolates to sequence types (STs) and clonal complexes (CCs), which are essential for accurate comparison with internationally reported lineages and for assessing their association with human listeriosis. Therefore, the absence of high-resolution sequence-based typing should be considered a limitation of the present study. Future investigations should incorporate MLST and/or WGS to better define the population structure and public health relevance of *L. monocytogenes* isolates recovered from smoked fish.

## 5. Conclusions

This preliminary retail survey indicates that *L. monocytogenes* contamination in smoked fish products was limited to cold-smoked, sliced Atlantic salmon, while no isolates were recovered from hot-smoked products. Importantly, all positive samples remained below 100 CFU/g, which is encouraging from a food-safety perspective. Given that Poland is an important producer and market for smoked fish products, these findings support the overall effectiveness of current production and hygiene controls, while also identifying cold-smoked salmon as a product category that warrants continued attention.

Although the detected levels were low, the recovered *L. monocytogenes* isolates carried key virulence determinants (LIPI-1 markers and internalins), and the complementary detection of stress- and biofilm-associated genes provided additional context on persistence potential in processing environments. Notably, the combination of culture-based detection, quantitative testing, multiplex PCR for virulence and serogrouping, PFGE, and antimicrobial susceptibility testing demonstrates that widely accessible laboratory methods can deliver substantial information on the potential pathogenicity, relatedness, and phenotypic susceptibility of isolates relevant to RTE food safety assessment. Such an approach can be implemented in routine monitoring and targeted investigations without requiring advanced sequencing infrastructure.

High-resolution genomic typing (e.g., WGS) would further enhance source attribution and discrimination of closely related strains; however, it is not universally available in all laboratories. Therefore, maintaining robust surveillance based on standardized, cost-effective methods remains highly relevant for risk management along the smoked fish supply chain, particularly at post-smoking steps such as slicing and packaging.

## Figures and Tables

**Table 1:** Characteristics of smoked fish samples included in the study.

Parameter	Category	Number of Samples (*n*)
Total samples		46
Fish species	Salmon (*Salmo salar*)	24
	Rainbow trout (*Oncorhynchus mykiss*)	9
	Mackerel (*Scomber scombrus*)	6
	Sprat (*Sprattus sprattus*)	4
	Herring (*Clupea harengus*)	1
	Lake trout (*Salvelinus namaycush*)	1
	Hake (*Merluccius merluccius*)	1
Processing type	Cold-smoked	13
	Hot-smoked	33
Origin of fish	Aquaculture	Majority
	Wild-caught	Minority
Packaging type	Vacuum-packed/modified atmosphere packaging (MAP)	Majority
Declared storage temperature	2–8 °C	All samples
Sampling source	Retail RTE products	46

**Table 2:** Gene classification, primer sequences (5′-3′), and PCR conditions used for detection of virulence, biofilm-associated, and stress response genes in *L. monocytogenes*.

Gene Classification	Gene Id	Primer Sequences (5′-3′)	Ref	Product (bp)	PCR Program (Modified In-House)
LIPI-1 regulon/virulence genes (multiplex PCR)	*hlyA*	F: ATCATCGACGGCAACCTCGGAGAC R: CACCATTCCCAAGCTAAACCAGTGC	[40,41]	404	Initial denaturation: 95 °C for 5 min; 40 cycles of 95 °C for 20 s, 58 °C for 40 s, 72 °C for 90 s; final extension: 72 °C for 7 min; hold at 4 °C.
	*prfA*	F: ACCAATGGGATCCACAAGA R: CAGCTGAGCTATGTGCGAT		467	
	*plcB*	F: AATATTTCAATCAATCGGTGGCTGA R: GGGTAGTCCGCTTTCGCTCTT		289	
	*actA*	F: CCAAGCGAGGTAAATACGGGA R: GTCCGAAGCATTTACCTCTTC		650	
Internalins—adhesion and invasion (multiplex PCR)	*inlB*	F: TGGGAGAGTAACCCAACCAC R: GTTGACCTTCGATGGTTGCT	[42]	884	Initial denaturation: 95 °C for 2 min; 30 cycles of 95 °C for 20 s, 55 °C for 20 s, 72 °C for 50 s; final extension: 72 °C for 2 min; hold at 4 °C.
	*inlJ*	F: TGTAACCCCGCTTACACAGTT R: AGCGGCTTGGCAGTCTAATA		238	
Internalins—adhesion (single PCR)	*inlA*	F: ACGAGTAACGGGACAAATGC R: CCCGACAGTGGTGCTAGATT	[42]	800	Initial denaturation: 95 °C for 2 min; 35 cycles of 95 °C for 20 s, 55 °C for 20 s, 72 °C for 75 s; final extension: 72 °C for 5 min; hold at 4 °C.
Biofilm-associated genes (single PCR)	*luxS*	F: ATGGCAGAAAAAATGAATGTAGAAA R: TTATTCACCAAACACATTTTTCCA	[43]	500	Initial denaturation: 95 °C for 3 min; 35 cycles of 95 °C for 30 s, 49 °C for 30 s, 72 °C for 1 min; final extension: 72 °C for 5 min; hold at 4 °C.
Biofilm-associated genes (single PCR)	*sigB*	F: TCATCGGTGTCACGGAAGAA R: TGACGTTGGATTCTAGACAC	[44]	320	Initial denaturation: 95 °C for 3 min; 35 cycles of 95 °C for 30 s, 52 °C for 30 s, 72 °C for 1 min; final extension: 72 °C for 5 min; hold at 4 °C.
Biofilm/Motility (single PCR)	*flaA*	F: TTACTAGATCAAACTGCTCC R: AAGAAAAGCCCCTCGTCC	[45]	538	Initial denaturation: 95 °C for 3 min; 35 cycles of 95 °C for 30 s, 51 °C for 30 s, 72 °C for 1 min; final extension: 72 °C for 5 min; hold at 4 °C.
Stress response/Adaptation (single PCR)	*gltA*	F: AAAGTGAGTTCTTACGAGATTT R: AATTAGGAAATCGACCTTCT	[45]	483	Initial denaturation: 95 °C for 3 min; 35 cycles of 95 °C for 30 s, 50 °C for 30 s, 72 °C for 1 min; final extension: 72 °C for 5 min; hold at 4 °C.

**Table 3:** Prevalence of *Listeria* spp. in examined smoked fish.

Product	In Total	No. (%) of Positive Samples		
		*Listeria* spp.	*L. monocytogenes*	*L. innocua*
Cold-smoked sliced salmon	12	5 (41.7%)	4 (33.3%)	1 (8.3%)
Cold-smoked salmon—fillet without skin	1	0	0	0
Hot-smoked salmon—fillet with skin	8	0	0	0
Hot-smoked salmon (small pieces of salmon meat, nests, rolls)	3	0	0	0
Hot-smoked rainbow trout (fillet with or without skin, whole fish)	6	0	0	0
Cold-smoked sliced rainbow trout	3	0	0	0
Hot-smoked mackerel (fillet with skin, gutted whole fish head-off)	6	0	0	0
Hot-smoked sprats	4	0	0	0
Hot-smoked: lake trout—fillet with skin, herring—fillet with skin, hake—whole fish, gutted, head-off)	3	0	0	0
In total	46	5 (10.9%)	4 (8.7%)	1 (2.2%)

**Table 4:** Summary of biochemical identification, molecular confirmation, and serogrouping results of *Listeria* isolates.

Isolate	Biochemical Identification	*lmo2234*	*prs*	*lmo0737*	*lmo1118*	*orf2110*	*orf2819*	Final Interpretation/Serogroup
R10	*L. monocytogenes*	+	+	+	−	−	−	serogroup IIa
R18	*L. monocytogenes*	+	+	−	+	−	−	serogroup IIc
R31	*L. monocytogenes*	+	+	+	−	−	−	serogroup IIa
R46	*L. monocytogenes*	+	+	−	+	−	−	serogroup IIc
R14	*L. innocua*	−	+	−	−	−	−	non-*L. monocytogenes*

Note: The symbols + and − indicate the presence and absence of the target gene, respectively.

**Table 5:** Distribution of virulence-associated and environmental adaptation genes among *Listeria* isolates.

Isolate	*prfA*	*hlyA*	*plcB*	*actA*	*inlA*	*inlB*	*inlJ*	*sigB*	*luxS*	*flaA*	*gltA*
R10	+	+	+	+	+	+	+	+	+	+	+
R18	+	+	+	+	+	+	+	+	+	+	+
R31	+	+	+	+	+	+	+	+	+	+	+
R46	+	+	+	+	+	+	+	+	+	+	+
R14	−	± *	–	−	−	−	−	+	+	+	+

* Note: The symbols +, −, and ± indicate the presence of the target gene, absence of the target gene, and an ambiguous result associated with faint non-specific amplification, respectively. A faint non-specific band was observed at the *hlyA* locus in isolate R14.

## Data Availability

The original contributions presented in this study are included in the article and Supplementary Materials. Further inquiries can be directed to the corresponding author.

## Supplementary Materials

The following supporting information can be downloaded at: https://www.mdpi.com/article/10.3390/foods15081406/s1, Table S1: Primer sequences used for molecular serogrouping of *Listeria monocytogenes* (multiplex PCR); Table S2: Biochemical identification of *Listeria* isolates using the Listeria System 18R; Table S3: Biochemical profiles and species identification using the VITEK^®^ 2 COMPACT system; Table S4: Selected genetic markers used to evaluate virulence, biofilm formation, and environmental adaptation in *L. monocytogenes*; Table S5: Antimicrobial susceptibility profiles of *L. monocytogenes* isolates recovered from RTE smoked fish samples. Figure S1: Species-specific PCR targeting the *lmo2234* marker; Figure S2: Multiplex PCR serogrouping of *Listeria* isolates according to the Doumith et al. method; Figure S3: Multiplex PCR detection of LIPI-1 virulence genes (*prfA*, *hlyA*, *plcB*, and *actA*) in *Listeria* isolates; Figure S4: Multiplex PCR detection of internalin genes (*inlA* and *inlJ*) in *Listeria* isolates; Figure S5: PCR detection of the *inlB* gene in *Listeria* isolates; Figure S6: PCR detection of stress response and quorum-sensing genes (*sigB* and *luxS*) in *Listeria* isolates.
