# Supplementary material for: Molecular Characterization, Virulence Profiling, and Antimicrobial Susceptibility of Listeria monocytogenes Isolated from Smoked Fish in Poland: A Preliminary Study
Source: Foods. 2026 Apr 17;15(8):1406. doi: 10.3390/foods15081406 (PMC13115546; doi:10.3390/foods15081406)
Supplement: Supplementary file 1 [file foods-15-01406-s001.zip › foods-4215240-supplementary.pdf]

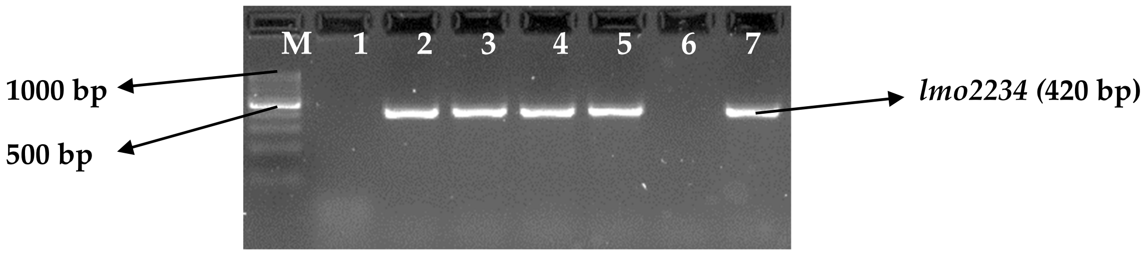

Figure S1. Species-specific PCR targeting the *lmo2234* marker (420 bp). Lane M: molecular weight marker; lane 1: *Listeria innocua* isolate R14 (negative for *lmo2234*); lanes 2–5: *Listeria monocytogenes* isolates (R46, R10, R18, R31) showing the expected amplicon; lane 6: no-template control (NTC); lane 7: positive control (*L. monocytogenes* ATCC 15313).

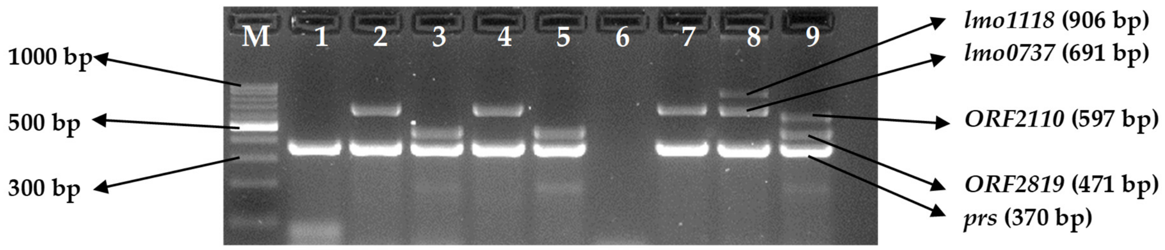

Figure S2. Multiplex PCR serogrouping of *Listeria* isolates according to the Doumith et al. method. Lane M: molecular weight marker; lane 1: *Listeria innocua* isolate R14 showing only the genus-specific *prs* amplicon; lanes 2 and 4: *L. monocytogenes* isolates R46 and R18 showing the *prs*, *lmo0737*, and *lmo1118* amplicons, corresponding to molecular serogroup IIc; lanes 3 and 5: *L. monocytogenes* isolates R10 and R31 showing the *prs* and *lmo0737* amplicons, corresponding to molecular serogroup IIa; lane 6: no-template control (NTC); lanes 7–9: reference strains, including serogroup IIa (*L. monocytogenes* ATCC 15313), serogroup IIc (*L. monocytogenes* ATCC 7644), and serogroup IVb (*L. monocytogenes* ATCC 19111).

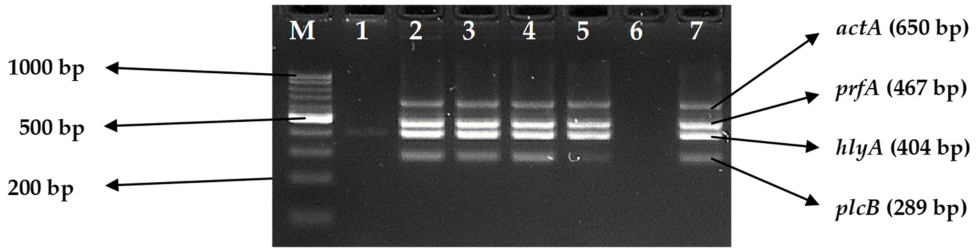

Figure S3. Multiplex PCR detection of LIPI-1 virulence genes (*prfA, hlyA, plcB, actA*) in *Listeria* isolates. Lane M: molecular weight marker; lane 1: *L. innocua* isolate R14 lacking the complete LIPI-1 profile, with only a faint non-specific band at the *hlyA* locus; lanes 2–5: *L. monocytogenes* isolates (R46, R10, R18, R31) showing all expected amplicons corresponding to the LIPI-1 gene cluster; lane 6: no-template control (NTC); lane 7: positive control (*L. monocytogenes* ATCC 15313).

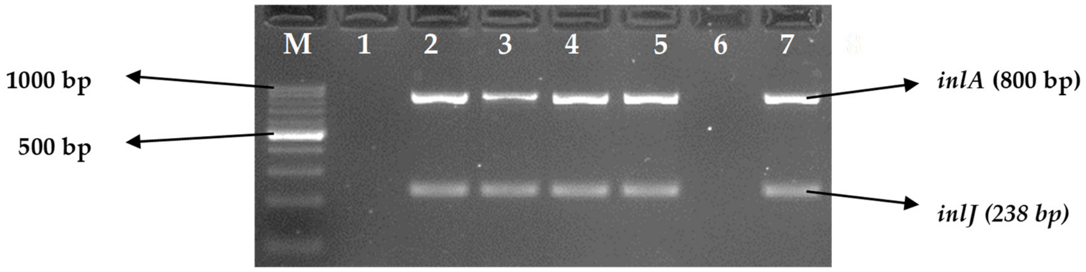

Figure S4. Multiplex PCR detection of internalin genes (*inlA* and *inlJ*) in *Listeria* isolates. Lane M: molecular weight marker; lane 1: *L. innocua* isolate R14 lacking internalin genes; lanes 2–5: *L. monocytogenes* isolates (R46, R10, R18, R31) showing the expected amplicons for both *inlA* and *inlJ*; lane 6: no-template control (NTC); lane 7: positive control (*L. monocytogenes* ATCC 15313).

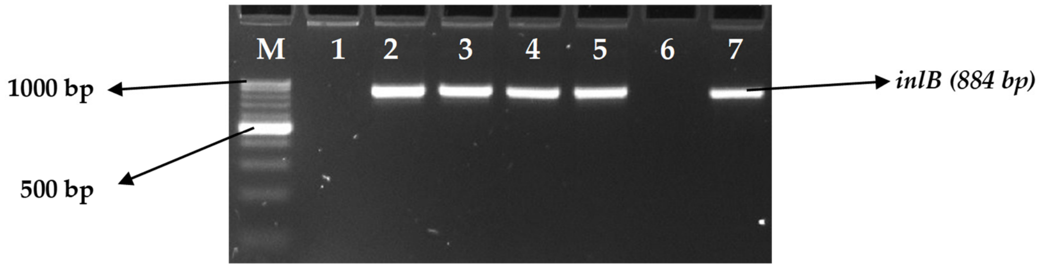

Figure S5. PCR detection of the *inlB* gene in *Listeria* isolates. Lane M: molecular weight marker; lane 1: *L. innocua* isolate R14 lacking *inlB*; lanes 2–5: *L. monocytogenes* isolates (R46, R10, R18, R31) showing the expected amplicon; lane 6: no-template control (NTC); lane 7: positive control (*L. monocytogenes* ATCC 15313).

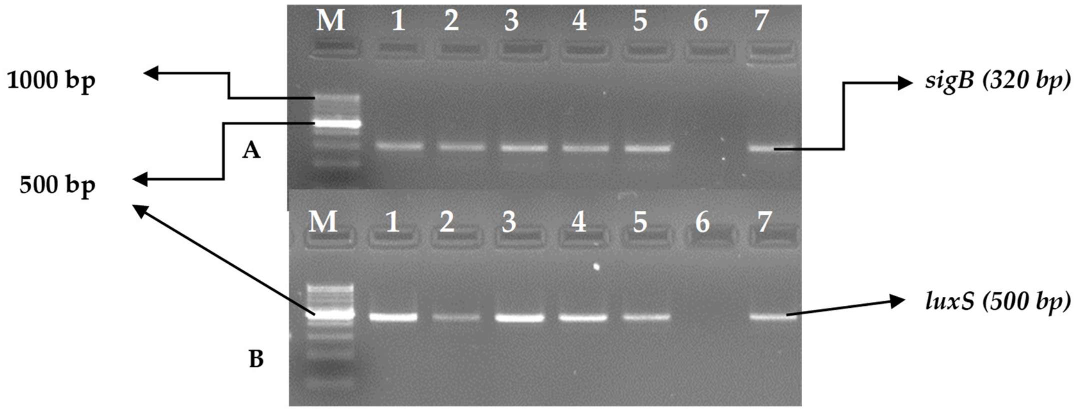

Figure S6. PCR detection of stress response and quorum-sensing genes (*sigB* and *luxS*) in *Listeria* isolates. Lane M: molecular weight marker; lane 1: *L. innocua* isolate R14; lanes 2–5: *L. monocytogenes* isolates (R46, R10, R18, R31) showing the expected amplicons for both *sigB* and *luxS*; lane 6: no-template control (NTC); lane 7: positive control (*L. monocytogenes* ATCC 15313).
