# Supplementary material for: Molecular Characterization, Virulence Profiling, and Antimicrobial Susceptibility of Listeria monocytogenes Isolated from Smoked Fish in Poland: A Preliminary Study
Source: Foods. 2026 Apr 17;15(8):1406. doi: 10.3390/foods15081406 (PMC13115546; doi:10.3390/foods15081406)
Supplement: Supplementary file 1 [file foods-15-01406-s001.zip › Supplementary_Table_S1.pdf]

Supplementary Table S1. Primer sequences used for molecular serogrouping of *Listeria monocytogenes* (multiplex PCR).

**Primer name**	**Sequence (5′→3′)**	**Product size (bp)**	**Reference**
lmo0737	F:AGGGCTTCAAGGACTTACCC<br>R:ACGATTTCTGCTTGCCATTC	691	[20]
lmo1118	F:AGGGGTCTTAAATCCTGGTT<br>R:CGGCTTGTTCGGCATACTTA	906	
orf2819	F:AGCAAAATGCCAAAACTCGT<br>R:CATCACTAAAGCCTCCCATTG	471	
orf2110	F:AGTGGACAATTGATTGGTGAA<br>R :CATCCATCCCTTACTTTGGAC	597	
prs (genus specific)	F:GCTGAAGAGATTGCGAAAGAAG<br>R:CAAAGAAACCTTGGATTTGCGG	370	
lmo2234	F: TGTCCAGTTCCATTTTTAACT<br>R: TTGTTGTTCTGCTGTACGA	420	[19]
