# Supplementary material for: Molecular Characterization, Virulence Profiling, and Antimicrobial Susceptibility of Listeria monocytogenes Isolated from Smoked Fish in Poland: A Preliminary Study
Source: Foods. 2026 Apr 17;15(8):1406. doi: 10.3390/foods15081406 (PMC13115546; doi:10.3390/foods15081406)
Supplement: Supplementary file 1 [file foods-15-01406-s001.zip › Supplementary_Table_S2.pdf]

Supplementary Table S2. Biochemical identification of *Listeria* isolates using the Listeria System 18R.

**Reaction (well)**	***L. monocytogenes* ATCC 19111**	**R 31**	**R 10**	**R 18**	**R 46**	**R 14**
ONPG	-	-	-	-	-	-
URE	-	-	-	-	-	-
H_2S	-	-	-	-	-	-
IND	-	-	-	-	-	-
RAF	-	-	-	-	-	-
CAT	+	+	+	+	+	+
MR	+	+	+	+	+	+
ESC	+	+	+	+	+	+
ABN	-	-	-	-	-	-
GLU	+	+	+	+	+	+
ARL	+	+	+	+	+	+
MAL	+	+	+	+	+	+
RAM	-	+	+	+	+	+
AMDM	+	-	+	+	+	+
XYL	-	-	-	-	-	+
MAN	-	-	-	-	-	-
NIT	-	-	-	-	-	-
VP	+	+	+	+	+	+
