# Supplementary material for: Molecular Characterization, Virulence Profiling, and Antimicrobial Susceptibility of Listeria monocytogenes Isolated from Smoked Fish in Poland: A Preliminary Study
Source: Foods. 2026 Apr 17;15(8):1406. doi: 10.3390/foods15081406 (PMC13115546; doi:10.3390/foods15081406)
Supplement: Supplementary file 1 [file foods-15-01406-s001.zip › Supplementary_Table_S3.pdf]

Supplementary Table S3. Biochemical profiles and species identification of Listeria isolates obtained using the VITEK® 2 COMPACT system (bioMérieux, Craponne, France).

**Reaction**	**R 10**	**R 18**	**R 31**	**R 46**	**R 14**
AMY	+	+	+	+	+
PIPLC	+	+	-	-	+
dXYL	-	-	-	-	-
ADH1	-	-	-	-	-
BGAL	-	-	-	-	-
AGLU	+	+	+	+	+
APPA	-	-	-	-	-
CDEX	+	+	+	+	+
AspA	-	-	-	-	-
BGAR	-	-	-	-	-
AMAN	-	-	+	+	-
PHOS	-	-	-	-	-
LeuA	-	-	-	-	-
ProA	-	-	-	-	-
BGURr	-	-	-	-	-
AGAL	-	-	-	-	-
PyrA	-	-	-	-	-
BGUR	-	-	-	-	-
AlaA	-	-	-	-	-
TyrA	+	+	-	-	+
dSOR	-	-	-	-	-
URE	-	-	-	-	-
POLYB	+	+	+	+	+
dGAL	-	-	-	-	-
dRIB	-	-	-	-	-
ILATk	-	-	-	-	+
LAC	-	-	-	-	+
NAG	+	+	+	+	+
dMAL	+	+	+	+	+
BACI	+	+	+	+	+
NOVO	+	+	+	+	+
NC6.5	+	+	+	+	+
dMAN	-	-	-	-	-
dMNE	+	+	+	+	+
MBdG	+	+	+	+	+
PUL	-	-	-	-	-
dRAF	-	-	-	-	-

**Reaction**	**R 10**	**R 18**	**R 31**	**R 46**	**R 14**
O129R	+	+	+	+	+
SAL	+	+	+	+	+
SAC	-	-	-	-	+
dTRE	+	+	+	+	+
ADH2s	-	-	-	-	-
OPTO	+	+	+	+	+
