# Supplementary material for: Molecular Characterization, Virulence Profiling, and Antimicrobial Susceptibility of Listeria monocytogenes Isolated from Smoked Fish in Poland: A Preliminary Study
Source: Foods. 2026 Apr 17;15(8):1406. doi: 10.3390/foods15081406 (PMC13115546; doi:10.3390/foods15081406)
Supplement: Supplementary file 1 [file foods-15-01406-s001.zip › Supplementary_Table_S4_.pdf]

Supplementary Table S4. Selected genetic markers used to evaluate virulence, biofilm formation, and environmental adaptation in *Listeria monocytogenes*.

**Stage of Infection / Biological Function**	**Gene**	**Encoded Protein**	**Molecular Mechanism and Role in Pathogenesis / Adaptation**	**References**
**Adhesion & Internalization**	*inlA*	Internalin A	Interacts with host E-cadherin; mediates transcytosis and crossing of the intestinal epithelial barrier.	[21-22]
	inlB	Internalin B	Interacts with the c-Met receptor tyrosine kinase; promotes invasion into a broad spectrum of host cells, including hepatocytes.	[23-24]
	inlJ	Internalin J	Sortase-anchored adhesin; validated biomarker for highly virulent lineages, crucial for *in vivo* survival and post-entry stages.	[25,42]
**Intracellular Survival & Spread (LIPI-1)**	*prfA*	PrfA	Pleiotropic transcriptional activator (CRP/FNR family); serves as the master switch regulating the core virulence gene repertoire.	[26-27]
	hlyA	Listeriolysin O (LLO)	Cholesterol-dependent cytolysin; mediates phagosomal membrane permeabilization and subsequent bacterial escape into the host cytosol.	[28-29]
	actA	ActA	Recruits the host Arp2/3 complex to induce focal actin polymerization, facilitating intracellular motility and cell-to-cell spread.	[30-31]
	plcB	Phospholipase C (PC-PLC)	Broad-spectrum phospholipase; required for the lysis of double-membrane secondary vacuoles during cell-to-cell spread.	[32,60]
**Biofilm Formation & Adaptation**	*luxS*	S-ribosylhomocysteinase	Involved in autoinducer-2 (AI-2) biosynthesis; mediates *quorum sensing* signaling that influences biofilm architecture and population dynamics.	[33,43]
	sigB	Sigma factor B	Alternative transcription factor; master regulator of the general stress response (e.g., osmotic, acid) exhibiting extensive cross-talk with the PrfA regulon.	[34-35]

Stage of Infection / Biological Function	Gene	Encoded Protein	Molecular Mechanism and Role in Pathogenesis / Adaptation	Refere nces
	flaA	Flagellin	Major structural component of the flagellum; essential for overcoming hydrodynamic barriers and initial attachment to abiotic surfaces.	[36-37]
**Metabolic Fitness**	*gltA*	Citrate synthase	Key enzyme of the TCA cycle; serves as a robust indicator of central carbon metabolism efficiency and overall metabolic fitness during environmental adaptation.	[38-39]
