# Supplementary material for: Molecular Characterization, Virulence Profiling, and Antimicrobial Susceptibility of Listeria monocytogenes Isolated from Smoked Fish in Poland: A Preliminary Study
Source: Foods. 2026 Apr 17;15(8):1406. doi: 10.3390/foods15081406 (PMC13115546; doi:10.3390/foods15081406)
Supplement: Supplementary file 1 [file foods-15-01406-s001.zip › Supplementary_Table_S5.pdf]

Supplementary Table S5. Antimicrobial agents, tested concentrations, and interpretive criteria used for MIC analysis of Listeria isolates.

Part A. Antimicrobial agents included in the 96-well MICRONAUT Special Plates panel and concentration ranges provided by the manufacturer (MERLIN Diagnostika GmbH, Bremen, Germany).

Antimicrobial agent (abbreviation)	Concentrations (µg/mL)
Amoxicillin (AMX)	0.25, 2, 4, 8, 16
Amoxicillin and clavulanic acid (AMX/CL)	4/2, 8/4, 16/8
Cephalexin (CFX)	8, 16
Cephapirin (CPH)	8
Ceftiofur (CFTI)	2
Cefquinome (CFQ)	2, 4
Cloxacillin (CLO)	2
Penicillin G (PG)	0.0625, 0.125, 2, 8
Nafcillin (NAF)	2
Gentamicin (GEN)	4, 8
Neomycin (NEO)	8
Streptomycin (STR)	8
Colistin (COL)	2
Enrofloxacin (ENR)	0.5, 2
Norfloxacin (NOR)	1, 2
Doxycycline (DOX)	2, 4, 8
Oxytetracycline (OXY)	2, 4, 8
Erythromycin (ERY)	0.25, 0.5
Florfenicol (FLR)	2, 4
Lincomycin (LIN)	2, 8
Lincomycin/spectinomycin (LIN/SP)	8, 32
Trimethoprim-sulfamethoxazole (TR/SMX)	2/38
Tiamulin (TIA)	16
Tylvalosin (TYLV)	2, 4

Abbreviations follow the naming given in the original supplementary source.

Part B. CLSI M45 interpretive criteria for Listeria monocytogenes.

Antibiotic	S	I	R
Penicillin	≤2	-	-
Ampicillin	≤2	-	-
Meropenem	≤0.25	-	-
Trimethoprim	≤0.5/9.5	-	-

S, susceptible; I, intermediate; R, resistant. For some organism/antimicrobial agent combinations, the absence or rare occurrence of resistant strains precludes defining result categories other than susceptible.

Part C. EUCAST interpretive criteria for Listeria monocytogenes.

Antibiotic	S	I	R
Benzylpenicillin	≤1	-	>1
Ampicillin	≤1	-	>1
Meropenem	≤0.25	-	>0.25
Erythromycin	≤1	-	>1
Trimethoprim	≤0.06	-	>0.06

S, susceptible; R, resistant. Erythromycin can be used to determine susceptibility to azithromycin and clarithromycin.

Part D. Tentative epidemiological cut-off values (TECOFF) according to EUCAST.

Antibiotic	Tentative MIC breakpoint (mg/L)
Benzylpenicillin	1
Ampicillin	1
Chloramphenicol	32
Daptomycin	8
Erythromycin	1
Gentamicin	2
Meropenem	0.5
Moxifloxacin	1
Piperacillin-tazobactam	8
Vancomycin	2
